# Relationships between academic performance of medical students and their workplace performance as junior doctors

**DOI:** 10.1186/1472-6920-14-157

**Published:** 2014-07-30

**Authors:** Sandra E Carr, Antonio Celenza, Ian B Puddey, Fiona Lake

**Affiliations:** 1 Faculty of Medicine, Dentistry and Health Sciences, The University of Western Australia, MB515, 35 Stirling Hwy, Crawley 6009, WA, Australia; 2 School of Primary, Aboriginal and Rural Health Care, Faculty of Medicine, Dentistry and Health Sciences, The University of Western Australia, Crawley, Australia; 3 School of Medicine and Pharmacology, Faculty of Medicine, Dentistry and Health Sciences, The University of Western Australia, Crawley, Australia

**Keywords:** Workplace based assessment, Junior doctors, Undergraduate medicine

## Abstract

**Background:**

Little recent published evidence explores the relationship between academic performance in medical school and performance as a junior doctor. Although many forms of assessment are used to demonstrate a medical student’s knowledge or competence, these measures may not reliably predict performance in clinical practice following graduation.

**Methods:**

This descriptive cohort study explores the relationship between academic performance of medical students and workplace performance as junior doctors, including the influence of age, gender, ethnicity, clinical attachment, assessment type and summary score measures (grade point average) on performance in the workplace as measured by the Junior Doctor Assessment Tool.

**Results:**

There were two hundred participants. There were significant correlations between performance as a Junior Doctor (combined overall score) and the grade point average (r = 0.229, P = 0.002), the score from the Year 6 Emergency Medicine attachment (r = 0.361, P < 0.001) and the Written Examination in Year 6 (r = 0.178, P = 0.014). There was no significant effect of any individual method of assessment in medical school, gender or ethnicity on the overall combined score of performance of the junior doctor.

**Conclusion:**

Performance on integrated assessments from medical school is correlated to performance as a practicing physician as measured by the Junior Doctor Assessment Tool. These findings support the value of combining undergraduate assessment scores to assess competence and predict future performance.

## Background

While we know the purpose of medical schools is to educate and train medical students in preparation for the role of junior doctor, the role of the medical student and the role of the junior doctor are different [1]. If assessment is matched to role, it may be difficult therefore, to assess medical students’ readiness to begin practice as a junior doctor, and to ensure that students who do not have the appropriate knowledge and skills do not progress [2]. In addition, there is a significant diversity in approach. Medical schools impart knowledge, provide opportunities to develop and practice skills, explore attitudes and apply behaviours relevant for practicing doctors. Many refer to such tools as the Australian Junior Doctor Curriculum Framework [3,4] and the UK General Medical Council (GMC) Tomorrow’s Doctors [5], for guidance as to what attributes a junior doctor needs. In addition, much effort is taken to reliably assess medical students on their knowledge and performance related to these outcomes, in attempts to ensure competence. However, medical school curricula are not standardised against such guidelines and neither are the assessment methods used [6]. Such a diversity of approaches is likely to add to a mismatch between measurement of undergraduate academic performance and workplace performance of junior doctors [7]. Studies of the predictive validity of undergraduate academic performance on workplace performance of junior doctors, in general have shown poor correlation between measures of performance at these two levels [8].

This poor correlation is supported by literature showing junior doctors do not always feel sufficiently prepared with respect to time management, aspects of prescribing and complex practical procedures, but feel most prepared for working with patients and colleagues, history taking and examination [9]. One large study of UK medical schools found the proportion who agreed they had been well prepared for practice (after one year) ranged from 30 to 82% [10]. They concluded that the vast knowledge base of clinical practice makes full preparation impossible. Many forms of assessment can be used to study a medical student’s knowledge or competence, however, if full preparation is not possible, undergraduate competence may not reliably predict performance in clinical practice [11]. Additionally, we know that the reliability and validity of assessments is context specific and there are reports suggesting the final examination is not related to a student’s clinical experiences, hence calling into question the validity of final examinations [12]. Differences in educational experiences in undergraduate Australian medical courses have been shown to influence how adequately prepared doctors are for their early working life [13]. Since these earlier studies, there have been changes in Australia and elsewhere in terms of both outcomes and assessment at both undergraduate and postgraduate levels. In addition, a shift to identifying poor performers rather than just determining a minimal standard (50%) suggests a broader view of how we identify who needs support and any action that should be taken. The purpose of this paper therefore is to explore the relationship between academic performance of medical students and workplace performance as junior doctors using a range of knowledge based, clinically based and combined measures, to see if they can be used to predict which students may need additional support.

## Methods

### Context

This descriptive cohort study, explores the relationships between assessment of academic performance of medical students and workplace performance of these medical students as junior doctors in the first postgraduate year (PGY1). Specifically, it explores whether students with lower scores in medical school also have lower scores as junior doctors and whether performance in medical school predicts performance in assessment as a junior doctor.

#### *Sample*

Two groups of students from the 5th and 6th year of a 6-year undergraduate medical curriculum (n = 302) at a single University were asked to consent to the collection of data about their undergraduate and early postgraduate assessment performance. The small number of students who failed, requiring them to repeat a year, could not be included as longitudinal data could not be obtained. Graduands seek employment, through application and interview, at a tertiary hospital of their choice to commence their first postgraduate year of work and training as a junior doctor. During this first year, the junior doctors rotate through five different, 10 week clinical terms. Ethics approval was obtained from the University of Western Australia as well as the three tertiary hospital settings in which the graduands worked for their first postgraduate year.

### Tool description

The Junior Doctor Assessment Tool (JDAT) has been developed to assess performance in clinical management, communication skills and professional behaviour throughout the first two postgraduate years. The tool consists of 10 discrete items that align to the areas of competency described in the Australian Junior Doctor Curriculum Framework. Each junior doctor is intended to be assessed summatively five times during their first postgraduate year (PGY1) [14], with the JDAT being completed by the supervising clinician at the end of each 10 week rotation or attachment.

In a validation study of the JDAT, we identified a Cronbach Alpha of 0.883 for the 10 item scale and identified that two principal components of junior doctor performance are being assessed rather than the commonly reported three [15]. Cronbach Alphas were 0.829 for the 6 item ‘Clinical Management subscale’ and 0.834 for the 4 item ‘Communication subscale’, indicating good internal consistency and reliability of the instrument in its entirety and for both subscales. It was asserted that professionalism was not assessed as a discrete entity; instead, professional behaviours were being assessed alongside or at the same time as assessing each of the items in the scale. For this reason, only the combined score, along with the scores of the two validated subscales have been used in this analysis.

### Dependent variables

The outcome variable of interest is the junior doctor performance in PGY1 measured using the JDAT, with the mean combined overall score (out of 40), the mean score for the Clinical Management subscale (out of 30) and the mean score for Communication skills subscale (out of 10).

### Independent variables

The undergraduate academic performance measures used were written examination scores in Year 5 (Science and Practice of Medicine - comprising 5 Modified Essay Questions and 5 Short Answer Questions and Year 6 (Science and Practice of Medicine comprising 100 extended matching questions and 10 short answer questions); 16 station objective structured clinical examination (OSCE) scores for Year 4 and Year 5; and scores from clinical attachments (combination of ratings of clinical knowledge, procedural skill and professional behaviour) in Year 6 that would replicate their clinical activities as junior doctors in PGY1 (medicine, surgery, psychiatry, and emergency medicine). Additionally, a combined score, the Grade Point Average (GPA), was included as an independent predictor variable.

The Grade Point Average (GPA) is a simple numerical index summarising academic performance in a course. At UWA the GPA is calculated by:

$$\text{GPA}=\frac{\text{sum}\left(\text{unit}\;\text{points}\;*\;\text{grade}\;\text{GPA}\right)}{\mathrm{sum}\left(\text{unit}\;\text{points}\right)}$$

with a range of GPA from 0 to 7 units.

Demographic variables of interest included age, gender and self-identified ethnicity (coded into Asian and Caucasian). Aboriginal students were not coded in this study as there was concern that anonymity of the student may not be maintained due to the small number of indigenous students in the cohort.

### Data collection methods

The data of participant performance as medical students were collected directly from the student administration repository of assessment scores. Junior doctor performance data were collected directly from the medical administration departments in the public hospitals where they were employed in the first postgraduate year. This data had not previously been collected and collated for analysis by the University of training hospitals prior to this study. Data were entered, coded and de-identified into SPSS V20 for statistical procedures over a two year period between 2008 and 2009.

### Analysis

Quantitative analysis procedures have been applied to the data with descriptive statistics performed for each of the assessment measures as medical students and junior doctors. The mean with standard deviation (SD) and median with interquartile range (IQR) were calculated to report the dispersion of results and the number of students with a result that was lower than the 25th percentile of GPA were recorded. The influence of the independent variables on the dependent variable of interest was explored through Pearson’s Correlation, ANOVA and linear regression analysis with Bonferroni adjustment using SPSS. The predictor variables of interest were age group (less or equal to 23 years or greater than 23 years) gender, ethnicity, the mark from year 6 clinical attachments (medicine, surgery, psychiatry and emergency medicine), GPA and assessment method (OSCE, written examination).

## Results

### Respondents

Of the 302 eligible medical students, 237 consented to participate in the study (78%). Of these, data were available for collection from 200 junior doctors over the two year period (84% of the consented participants). The mean age of participants at the commencement of the study was 23 years (SD 2.3, range 20–37 years). The demographics of the respondents were representative of the population of the graduands with 54% of them being females, 104 (52%) self- identifying as Caucasian, 66 (33%) as Asian and 15% did not identify an ethnic background. There were no significant differences identified in the descriptive scores of academic performance for the two cohorts of respondents, or for the workplace performance scores for the three tertiary hospital training settings. Therefore the findings of both cohorts and all hospitals are reported together.

### Descriptive findings

As documented in Table 1, for the assessment of Year 6 clinical attachments, Psychiatry had the lowest mean scores and the largest standard deviation and Medicine demonstrated the highest mean score with the smallest standard deviation. Emergency Medicine had the fewest students with scores below the 25th percentile. For examinations, the Year 4 OSCE had the greatest number of students performing below the 25 percentile and the Year 6 Written exam had the least. The proportion of participants with scores below the 25th percentile as medical students (GPA) were similar in PGY1 (Combined score on JDAT).

**Table 1:** Measures of central tendency and dispersion for assessment measures

**Assessment Method (n = 200)**		**Mean (SD)**	**Median (IQR)**	**<25 percentile (count)**
***Assessment of clinical attachments year 6***	*Medicine*	*73.7 (5.1)*	*74 (71,77)*	*50*
	*Surgery*	*72.1(7.4)*	*73 (69,76)*	*52*
	*Emergency Medicine*	*73.3 (5.6)*	*71 (65, 78)*	*23*
	*Psychiatry*	*71.6 (8.6)*	*72 (65, 78)*	*42*
***Examinations***	*Year 4 OSCE*	*68.7 (8.2)*	*70 (65,74)*	*62*
	*Year 5 OSCE*	*66.3 (7.3)*	*67 (63, 71)*	*46*
	*Year 5 Written*	*68.3(8.3)*	*69 (63,73)*	*48*
	*Year 6 Written*	*71.1 (4.5)*	*71 (68, 74)*	*38*
***Summary scores***	*GPA*	*5.6 (.47)*	*5.6 (5.32, 5.93)*	*48*
***Assessment as a junior doctor***	*Clinical Management subscale (/24)*	*19.9 (1.37)*	*19.75 (19, 21)*	*40*
	*Communication skills subscale (/16)*	*14.4 (1.4)*	*14.3 (13.8, 15)*	*50*
	*Combined Overall Score (/40)*	*34.4 (2.23)*	*34.25 (33,36)*	*42*

### Effect of demographics

Females obtained higher mean scores than males for the Year 5 written examination (P = 0.001) and Emergency Medicine (P = 0.034) and Asian students obtained higher scores in the Year 6 Emergency Medicine (P = 0.030). In students older than 23 years there was a non-significant trend for higher mean scores on the Clinical Management subscale of the JDAT (P = 0.06) and significantly higher scores in the Year 5 OSCE examination (P = 0.045). There were no other significant effects of age, gender or ethnicity on measures of undergraduate performance or workplace performance.

### Relationships between academic and workplace assessment

As summarised in Table 2, there were significant correlations between performance as a Junior Doctor (combined overall score) and the GPA (r = 0.229, P = 0.002), the score from the Emergency Medicine attachment (r = 0.361, P = 0.000) and the Written Examination in Year 6 (r = 0.178, P = 0.014). These correlations persisted when the Clinical Management subscale and the Communication skills subscales were used suggesting that junior doctor performance in all assessed areas were related to these measures of undergraduate academic performance. Additionally, a significant correlation was observed between both the JDAT combined overall score and the score obtained on the Clinical Management subscale of the JDAT with both the Year 4 and Year 5 OSCE scores. There were significant correlations between the written assessment in Year 6 and workplace performance as measured by both the Clinical Management subscale (r = 0.136, P = 0.027) and the Combined Overall score on the JDAT (r = 0.178, P = 0.014).

**Table 2:** Correlations between academic measures and PGY1 performance

		**Junior Doctor Performance Measures**		
**Academic performance measures N = 200**		**Clinical management subscale**	**Communication subscale**	**Combined overall score**
GPA	r	0.280	0.288	0.257
	P	<0.001**	0.257	<0.001**
Year 4 OSCE	r	0.197	0.225	0.137
	P	0.003**	0.001**	0.027*
Year 5 OSCE	r	0.172	0.224	0.161
	P	0.007**	0.001**	0.022*
Emergency medicine	r	0.219	0.137	0.361
	P	0.001**	0.027*	<0.001**
Psychiatry	r	0.075	0.161	0.094
	P	0.146	0.011*	0.092
Medicine	r	0.194	0.157	0.162
	P	0.003**	0.013*	0.011*
Surgery	r	.026	0.091	.100
	P	0.358	0.100	0.080
Yr 5 written exam	r	0.153	0.067	0.076
	P	0.017*	0.177	0.148
Yr 6 written exam	r	0.136	0.088	0.178
	P	0.027*	0.107	0.014*

r = Pearson correlation coefficient, P = 2-tailed probability where *is significant at p < 0.05 and **is significant at p < 0.01.

### Effect of independent variables

In the final linear regression models there was no evidence of multicollinearity (the independent variables are not related), with tolerance statistics for all independent variables greater than 0.7 and the VIF values between 1.1 and 1.4. In regression analysis there was no significant effect for the demographic variables of age, gender or ethnicity. There was a significant effect of the method of assessment in medical school on the overall combined score of the junior doctor (F = 3.003, P = 0.042) but there were no significant Beta coefficients for any individual assessment method. As illustrated in Table 3, scores in Emergency Medicine attachments in medical school demonstrated a significant influence on overall combined scores on the JDAT (P < 0.001), however while this significant influence of Year 6 Clinical Attachments did persist for the Clinical Management subscale (F = 3.605, P = 0.007) it did not persist for the Communication subscale (F = 2.826, P = 0.26) . As depicted in Table 3, when measures of academic performance in medical school were summarised using GPA, a significant effect on the overall combined score of the junior doctor was observed (F = 14.080, P = 0.001) which persisted for both the Clinical Management subscale (F = 16.879, P < 0.000) and Communication subscale (F = 18.060, p < 0.000) indicating that performance in Emergency Medicine attachments or overall GPA both predicted performance in the junior doctor assessments used in PGY1.

**Table 3:** Multivariate linear regression and ANOVA of Combined Overall JDAT Score (workplace performance) with undergraduate clinical attachment scores, GPA or examination methods as the predictor variables

**Predictor variables**	**Category (n = 200)**	**Standardized coefficient beta (P value)**	**ANOVA F (P value)**
*Year 6 clinical attachments*	Medicine	0.104 (0.162)	2.846 (0.025)
	Surgery	0.077 (0.283)	
	Emergency Medicine	0.355 (<0.001)	
	Psychiatry	0.033 (0.633)	
*Method of assessments*	Year 5 Written	0.041 (0.859)	3.003 (0.042)
	Year 6 Written	0.105 (0.163)	
	Year 4 OSCE	0.060 (0.161)	
	Year 5 OSCE	0.123 (0.141)	
*Summary of academic performance*			
	GPA	0.251 (0.001)	14.08 (<0.001)

## Discussion

Medical students in this study, all of whom formally passed, demonstrated a range of scores in both examination and performance in clinical placements as undergraduates. Emergency Medicine attachments in medical school demonstrated a significant association with overall combined scores on the JDAT which persisted for the Clinical Management subscale but not the Communication subscale. The most significant effect of measures of academic performance on the overall combined score of the junior doctor was observed for the GPA and this persisted for both the Clinical Management subscale and Communication subscale. Others have found similar results [8], supporting the combination of assessments to produce the strongest predictive validity. It is well recognised that clinical practice as such requires competence in a range of attributes; therefore no single method of assessment is likely to provide enough data to make a valid and reliable judgement of this integrated competence [16].

Surprisingly there was only a small significant effect of the method of assessment in medical school (P = 0.042) on the overall combined score of the junior doctor. This effect was not explained by any individual assessment. There was a significant correlation between junior doctor performance and the Year 4 and 5 OSCE’s, which was amplified between the OSCE score and the Communication subscale suggesting the OSCE may have played some part in prediction of performance in workplace based assessment. In an OSCE, a student must demonstrate behaviours and knowledge but a ward assessment by a supervising consultant may inform the highest predictor of clinical competence - the combined implementation of medical knowledge, procedural skills, communication and professional behaviour. Most medical schools have an OSCE as part of their final barrier examination and [6] others have reported its ability to predict future performance in clinical and psychomotor skills [17,18]. However, many of these studies have not looked at junior doctor performance. Rather they have studied performance in pre-clinical examinations to predict clinical performance later in medical school. Results are mixed with other studies finding OSCE’s are not predictive of future performance post-graduation unless part of a comprehensive assessment process [8]. These findings again re-enforce the differences between the narrowed expected performance of medical students and broader integrated expectations of medical graduates and as such warrant further large scale, multi-centre research.

Why the emergency medicine attachment was the only clinical attachment predictive of performance as a junior doctor is interesting. The tools used in all clinical attachments in Year 6 are similar to those used in PGY1. Although the assessors in the emergency medicine attachment have not received additional training in assessment techniques, there are unique differences in the learning opportunities and the organisation of the clinical team compared to other ward and outpatient based clinical attachments. With a high turnover of patients presenting diagnostic and acute management problems, there are many opportunities for consolidating clinical and procedural skills. In addition, such skills need to be integrated in the immediate assessment and care of patients, which may be more closely aligned to those assessed in PGY1. Emergency medicine consultants who perform the assessments on medical students and junior doctors, spend much of their time on duty supervising in the clinical environment, as opposed to medicine or surgery where work on the wards (such as during ward rounds or emergency reviews) is intermittent. In addition, close working relations between Emergency Medicine team members may foster good communication about poor performance by students or junior doctors. This in turn may lead to a more accurate assessment of a person’s ability, with greater observation by more people. Such an approach may also provide more of a participatory learning environment, [19] enabling students and junior doctors opportunity, under close supervision, to engage and implement medical knowledge, thereby developing competence. Supporting our findings, one recent study has also identified emergency medicine as the clinical attachment in the early postgraduate years most likely to detect underperformance in junior doctors [20].

Limitations of the study are that there was a small loss to follow up for the cohort, only students who have passed were included and it is not known whether students had repeated any year in their course. The narrow inter-quartile range of scores for some items on the JDAT may limit the ability to interpret the findings. However, this was the only tool being used to assess workplace performance at the time of the study and it is still in use. While these limitations may affect the ability to generalise the findings prospectively to student groups and to other similar settings, at the time of publication there are no similar studies being undertaken within Western Australia.

## Conclusion

Research on assessment in medical education has been described as focusing on individual measurement instruments and their psychometric quality [21]. Despite its importance, predictive validity is a characteristic of assessment that is often neglected because of difficulties in the accurate determination of outcome. The findings of this current research support the value of combining undergraduate assessment scores to assess competence as a whole in predicting future performance. This is in line with support in recent years to develop programmatic approaches to assessment where a purposeful arrangement of methods is applied to measure competence comprehensively [21,22].

Adding to this is the knowledge that assessment of academic performance in medical school is not always aligned with assessing the generic graduate outcomes expected of the junior doctor in the workplace. If we adopt an approach to help students most likely to struggle, rather than narrowly using assessment to determine the failures, we can consider how the lower performers can be tracked, monitored, supported and remediated during the final year of medical school and through the first post graduate year of medical practice. How this more constructive approach can be best achieved needs to be explored and developed in collaboration between the higher education and postgraduate training providers.

## Competing interests

The authors declare that they have no competing interests.

## Authors’ contributions

This work was completed by four authors: SEC, AC, IP and FL. SEC was responsible for the study conception and design. SEC performed the data collection and data analysis. SEC was responsible for the drafting of the manuscript. AC, IP and FL made critical revisions to the paper. All authors read and approved the final manuscript.

## Pre-publication history

The pre-publication history for this paper can be accessed here:

http://www.biomedcentral.com/1472-6920/14/157/prepub
